# Harmonization of Next-Generation Sequencing Procedure in Italian Laboratories: A Multi-Institutional Evaluation of the SiRe® Panel

**DOI:** 10.3389/fonc.2020.00236

**Published:** 2020-03-11

**Authors:** Umberto Malapelle, Francesco Pepe, Pasquale Pisapia, Roberta Sgariglia, Mariantonia Nacchio, Caterina De Luca, Rosanna Lacalamita, Stefania Tommasi, Rosamaria Pinto, Grazia Palomba, Giuseppe Palmieri, Davide Vacirca, Massimo Barberis, Irene Bottillo, Paola Grammatico, Lucia Rosalba Grillo, Valerio Costa, Riccardo Smeraglio, Dario Bruzzese, Giancarlo Troncone

**Affiliations:** ^1^Department of Public Health, University of Naples Federico II, Naples, Italy; ^2^Molecular Diagnostics and Pharmacogenetics Unit, IRCCS Istituto Tumori “Giovanni Paolo II,”, Bari, Italy; ^3^Unit of Cancer Genetics, Institute of Biomolecular Chemistry (ICB), National Research Council (CNR), Sassari, Italy; ^4^Pathology Unit, European Institute of Oncology, Milan, Italy; ^5^Laboratory of Medical Genetics, Department of Molecular Medicine, San Camillo-Forlanini Hospital, Sapienza University, Rome, Italy; ^6^Department of Pathology, San Camillo-Forlanini Hospital, Sapienza University, Rome, Italy; ^7^Institute of Genetics and Biophysics (CNR), Naples, Italy

**Keywords:** colon cancer, lung cancer, predictive molecular pathology, next-generation sequencing, biomarkers

## Abstract

**Background:** Next-generation sequencing (NGS) needs to be validated and standardized to ensure that cancer patients are reliably selected for target treatments. In Italy, NGS is performed in several institutions and harmonization of wet and dry procedures is needed. To this end, a consortium of five different laboratories, covering the most part of the Italian peninsula, was constituted. A narrow gene panel (SiRe®) covering 568 clinically relevant mutations in six different genes (*EGFR, KRAS, NRAS, BRAF, cKIT*, and *PDGFR*α) with a predictive role for therapy selection in non-small cell lung cancer (NSCLC), gastrointestinal stromal tumor, colorectal carcinoma (CRC), and melanoma was evaluated in each participating laboratory.

**Methods:** To assess the NGS inter-laboratory concordance, the SiRe® panel, with a related kit and protocol for library preparation, was used in each center to analyze a common set of 20 NSCLC and CRC routine samples. Concordance rate, in terms of mutation detected and relative allelic frequencies, was assessed. Then, each institution prospectively analyzed an additional set of 40 routine samples (for a total of 160 specimens) to assess the reproducibility of the NGS run parameters in each institution.

**Results:** An inter-laboratory agreement of 100% was reached in analyzing the data obtained from the 20 common sample sets; the concordance rate of allelic frequencies distribution was 0.989. The prospective analysis of the run metric parameters obtained by each center locally showed that the analytical performance of the SiRe® panel in the different institutions was highly reproducible.

**Conclusions:** The SiRe® panel represents a robust diagnostic tool to harmonize the NGS procedure in different Italian laboratories.

## Introduction

In this era of precision oncology, predictive molecular pathology is key to assess actionable genetic targets in cancer patients (1–3). Thus, a large and steadily increasing number of predictive biomarkers need to be taken into account to personalize the therapeutic strategy in different solid tumors and in different patients (1–3). As an example, patients with metastatic colorectal cancer (mCRC), whose tumors are mutated in exons 2-3-4 of either Kirsten rat sarcoma (*KRAS*) or of neuroblastoma RAS viral oncogene homolog (*NRAS*) genes, are not eligible for target therapy with monoclonal antibodies against epidermal growth factor receptor (EGFR) protein (4–7). In addition, in mCRC, the National Comprehensive Cancer Network (NCCN) guidelines (Version 2.2018) recommend to genotype the patients for v-Raf murine sarcoma viral oncogene homolog B (*BRAF*) mutations, whose adverse prognostic role is well-established (8). Similarly, in non-small cell lung cancer (NSCLC) patients, the updated molecular testing guideline issued by the College of American Pathologists, the International Association for the Study of Lung Cancer, and the Association for Molecular Pathology defines *EGFR*, anaplastic lymphoma kinase (*ALK*), and ROS proto-oncogene 1 receptor tyrosine kinase as the “must test” genes to select patients for treatment with tyrosine kinase inhibitors (9–13). Even more recently, the American Society of Clinical Oncology established that also *BRAF* needs to be tested in all patients with advanced NSCLC as a positive predictive biomarker (14). In this rapidly evolving scenario, several are the technologies adopted to perform a molecular analysis. Among these, next-generation sequencing (NGS) represents a fascinating and versatile technology which is able to simultaneously analyze mutational hotspots in different gene targets for different cancer patients. However, only a laboratory with skilled and experienced personnel can reliably validate and implement NGS; moreover, the DNA quality and quantity derived from formalin-fixed and paraffin-embedded samples can be suboptimal and the number of samples classified as “inadequate” for molecular analysis is not negligible (15–19). As a result, when mutated alleles are only present in the subclonal neoplastic population, NGS might yield results which are not entirely consistent among the different institutions (20, 21).

To date, a large number of gene panels are commercially available for different clinical purposes; however, most of these panels are quite large and their use in routine practice is not cost-effective. Conversely, smaller gene panels seems to be more suitable than larger panels, especially when DNA input is less abundant and when the tested samples are represented by small tissue biopsies, as it often occurs in metastatic NSCLC patients (17, 18). To meet these challenges, in a previous study we have designed, developed, and validated, for both tissue samples and liquid biopsy specimens, a narrow NGS gene panel (SiRe®) that covers 568 clinically relevant mutations in six genes (*EGFR, KRAS, NRAS, BRAF, cKIT*, and *PDGFR*α) involved in NSCLC, gastrointestinal stromal tumor, mCRC, and melanoma (19–23).

In the current study, we aim to evaluate the performance of the SiRe® NGS panel in a multi-institutional study, thanks to a consortium constituted by five different Italian laboratories experienced in the application of NGS in a predictive molecular pathology setting.

## Materials and Methods

### SiRe® Gene Panel

**Figure 1:**
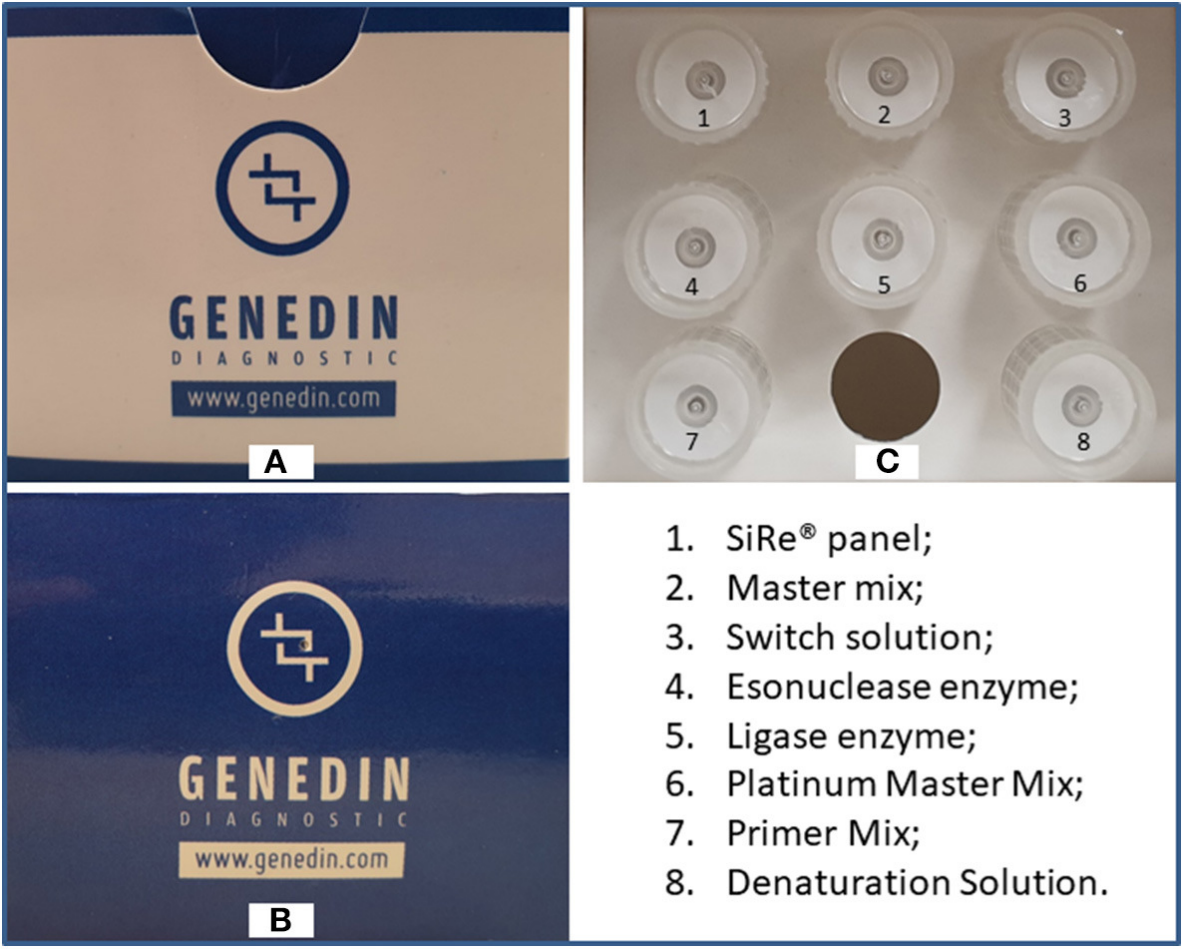
Upper **(A)** and frontal **(B)** parts of the SiRe® kit box and the related reagent tubes distributed in the internal **(C)** part. Permission to publish the figure was obtained by Genedin s.r.l. (a spin-off of the Department of Public Health, University of Naples Federico II).

**Figure 2:**
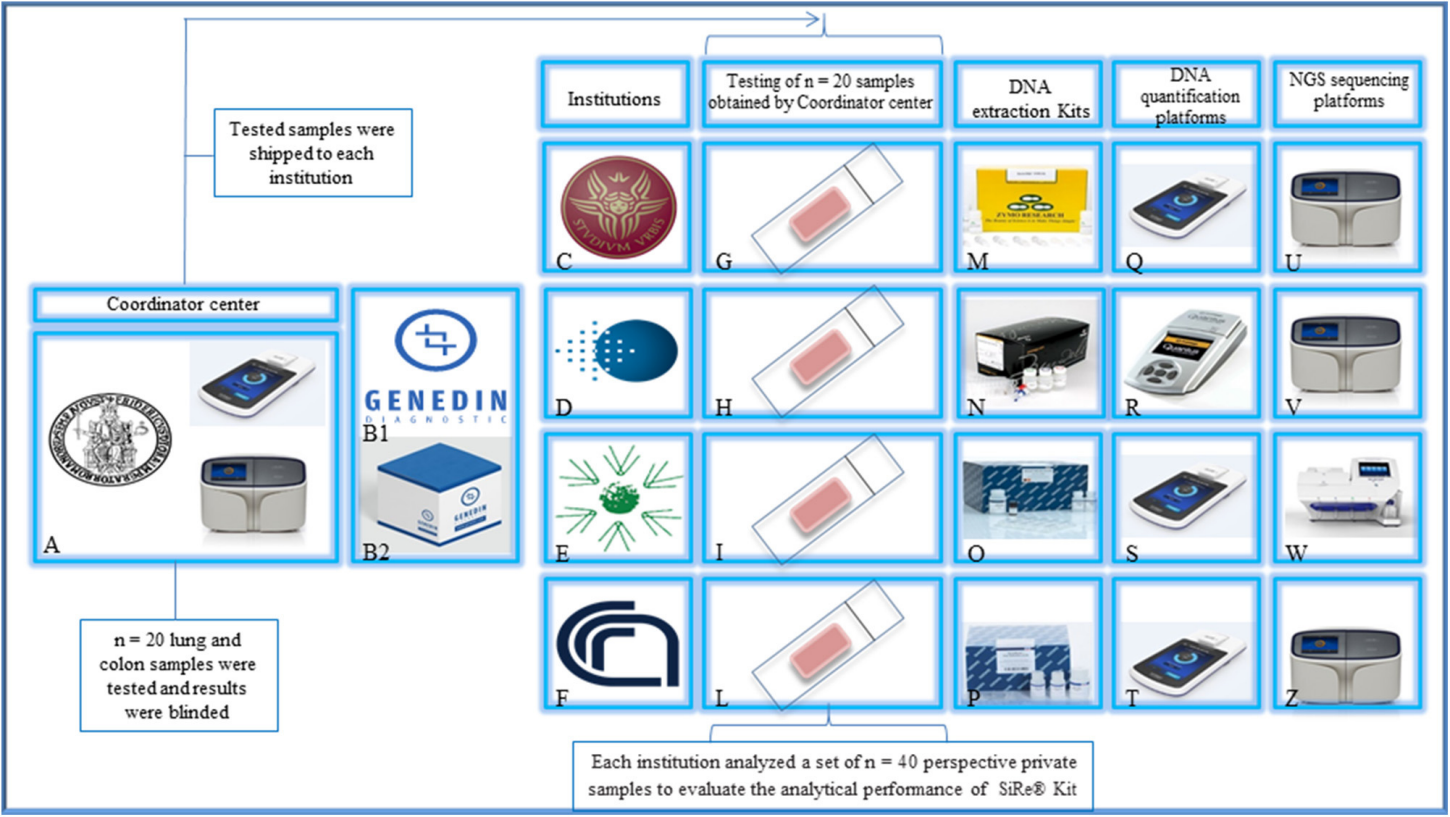
Study design. The Department of Public Health of the University of Naples Federico II **(A)** and Genedin s.r.l. (a spin-off of the Department of Public Health, University of Naples Federico II) **(B1)** develop the SiRe® NGS panel kit **(B2)**. The kit was distributed to *n* = 4 different institutions [**(C)** University La Sapienza—Rome, **(D)** Istituto Oncologico Europeo—Milan, **(E)** Istituto Tumori Giovanni Paolo II—Bari, **(F)** Consiglio Nazionale delle Ricerche—Sassari] to analyze a series of *n* = 160 colon/lung routine samples **(G–L)** (*n* = 40 for each institution) following the local NGS workflow, for DNA extraction **(M–P)**, quantification **(Q–T)**, and sequencing on IonTorrent platforms **(U–Z)**, after an alignment phase on a common set of *n* = 20 shared samples. Permission to publish the figure was obtained by Genedin s.r.l. (a spin-off of the Department of Public Health, University of Naples Federico II).

The SiRe® NGS panel was developed by the Department of Public Health of the University of Naples Federico II (Fed II) to assess 568 mutations in six different genes (*EGFR, KRAS, NRAS, BRAF, cKIT*, and *PDGFR*α) as previously described (Figure 1). This panel, together with all reagents and a dedicated protocol (Supplementary File 1) required to produce gene libraries, was distributed to four different institutions (Figure 2).

### Study Design

This study was designed to evaluate the multi-institutional performance of the SiRe® gene panel-based NGS assay (Supplementary File 1) by both a retrospective and a prospective analysis. The retrospective analysis verified the concordance rate among the different institutions (inter-laboratory concordance, Table 1), whereas the prospective part evaluated the SiRe® gene panel-based NGS assay performance in the routine daily practice.

**Table 1:** Mutations detected on a common sample set (*n* = 20) by the University of Naples Federico II with relative allelic frequencies and relative results obtained among all the participating institutions.

	**Fed II**			**Participating institutions**		
**Sample**	**Gene**	**Mutation**	**Frequencies (%)**	**Gene**	**Mutation**	**Frequencies (%)**
1	*PIK3CA*	p.R88Q	15.9	*PIK3CA*	p.R88Q	15.9; 15.9; 15.6; 15.8
	*PDGFRα*	p.V824V	47.8	*PDGFR*	p.V824V	47.1; 51.2; 47.7; 42.6
2	*KRAS*	p.G12C	18.9	*KRAS*	p.G12C	18.9; 16.8; 22.8; 18.9
3	*KRAS*	p.G12V	42.0	*KRAS*	p.G12V	41.3; 39.9; 37.1; 42.3
4	*NRAS*	p.G12D	36.1	*NRAS*	p.G12D	38.9; 36.3; 39.8; 34.9
5	*KRAS*	p.A146T	31.0	*KRAS*	p.A146T	31.1; 31.1; 23.7; NA
	*KIT*	p.M541L	53.4	*KIT*	p.M541L	53.1; 53.1; 47.1; NA
6	*KRAS*	p.G13D	57.9	*KRAS*	p.G13D	57.4; 57.4; 58.0; 65.3
7	*KRAS*	p.G12C	36.4	*KRAS*	p.G12C	35.1; 35.1; 40.5; 32.4
	*KIT*	p.M541L	58.4	*KIT*	p.M541L	58.4; 58.4; 60.3; 58.3
8	*KRAS*	p.G12D	18.1	*KRAS*	p.G12D	18.4; 18.4; 18.7; 21.8
	*PIK3CA*	p.E545K	11.0	*PIK3CA*	p.E545K	11.5; 11.5; 9.9; 9.3
	*KIT*	p.M541L	100.0	*KIT*	p.M541L	99.8; 99.8; 100.0; 99.7
9	*KRAS*	p.G12V	22.0	*KRAS*	p.G12V	22.3; 22.3; 24.0; 22.0
10	*KRAS*	p.G12V	11.0	*KRAS*	p.G12V	11.0; 11.0; 7.5; 9.3
	*PIK3CA*	p.E542K	10.8	*PIK3CA*	p.E542K	10.7; 10.7; 11.8; 10.5
11	*KRAS*	p.G12D	14.9	*KRAS*	p.G12D	14.4; 19.3; 17.6; 15.7
12	–	WT	–	–	WT	–
13	*KRAS*	p.G12S	27.8	*KRAS*	p.G12S	27.1; 31.5; 27.0; 36.5
14	*PDGFRα*	p.V824V	99.1	*PDGFR*	p.V824V	99.1; 99.2; 99.1; 99.4
15	–	WT	–	–	WT	–
16	*KIT*	p.M541L	50.4	*KIT*	p.M541L	46.8; 51.2; 50.3; 54.0
17	*KRAS*	p.G13D	45.2	*KRAS*	p.G13D	44.6; 46.5; 45.3; 46.2
	*PDGFR*	p.V824V	18.9	*PDGFR*	p.V824V	19.7; 13.7; 18.4; 20.6
18	*KRAS*	p.G12A	6.1	*KRAS*	p.G12A	6.3; 5.8; 5.5; 6.2
	*PDGFRα*	p.V824V	45.8	*PDGFR*	p.V824V	43.9; 47.6; 46.5; 48.1
19	–	WT	–	–	WT	–
20	*EGFR*	p.E746_S752>V	43.8	*EGFR*	p.E746_S752>V	45.4; 51.1; 45.5; 40.9

*BRAF, V-Raf murine sarcoma viral oncogene homolog B1; EGFR, epidermal growth factor receptor; KIT, KIT proto-oncogene, receptor tyrosine kinase; KRAS, V-Ki-Ras2 Kirsten rat sarcoma 2 viral oncogene homolog; NA, not assessed; NRAS, neuroblastoma RAS viral (V-Ras) oncogene homolog; PDGFRα, platelet-derived growth factor; PIK3CA, phosphatidylinositol-4,5-bisphosphate 3-kinase catalytic subunit alpha; WT, wild type; Fed II, University of Naples Federico II. For the participating institutions, the frequencies are reported from left to right: University La Sapienza—Rome, Istituto Oncologico Europeo—Milan, Consiglio Nazionale delle Ricerche—Sassari, Tumori Giovanni Paolo II—Bari*.

Briefly, the coordinating center (University of Naples Federico II) selected from its archives a set of 20 colon or lung cases; this set of cases was blinded and dispatched to the participating institutions that provided the coordinator center with the run metric parameters and with the complete list of all the mutations detected and their relative allelic fraction (AF) by 10 working days. Signal processing, base calling, and coverage analysis were carried out in a blinded way in each institution by using the SiRe® bed files on the Torrent Suite (Thermofisher). Variants were automatically annotated using a variant caller plug-in at specific optimized parameters of the SiRe® panel, as previously reported (19). The obtained BAM files were also visually inspected by an experienced user on the Golden Helix Genome Browser v.2.0.7 (Bozeman, MT, USA). Details relative to the DNA extraction procedures and to the NGS platform employed are reported in the Supplementary Table 1.

To assess the SiRe® gene panel-based NGS assay performance in the routine daily practice, each institution analyzed a distinct set of 40 colon or lung cancer tissue samples (Supplementary Table 2), reporting the analytical success rate, the median number of reads for the sample, the median read length, the median number of mapped reads, the percentage of reads on target, the average reads for amplicon, and the uniformity of coverage. Data were compared with the previously obtained “in-house” validation data set from the coordinator center to assess the analytical performance of the SiRe® kit in different clinical settings.

All of the analyzed cases were reviewed by experienced pathologists and featured at least 20% of neoplastic cells.

Written informed consent was obtained from all patients and documented in accordance with the general authorization to process personal data for scientific research purposes from “The Italian Data Protection Authority” (http://www.garanteprivacy.it/web/guest/home/docweb/-/docwebdisplay/export/2485392). All information regarding human material was managed using anonymous numerical codes, and all samples were handled in compliance with the Helsinki Declaration (https://www.wma.net/fr/news-post/en-matiere-de-transfert-des-taches-la-securite-des-patients-et-la-qualite-des-soins-devraient-etre-primordiales/). According to the aforementioned national guidelines, the double-blinded study did not require an Ethical Committee approval since it did not affect the clinical management of the involved patients' samples.

### Data Analysis

The mutations and their relative allelic frequencies concordance rate were assessed by using an intra-class correlation coefficient (ICC), while concordance between each institution and Federico II was evaluated using the Linn's concordance correlation coefficient (CCC) and further explored using Bland-Altman plots. CCC is a reproducibility index which allows assessing both precision and accuracy by evaluating the degree to which individual pairs fall on the line of perfect concordance (i.e., the 45° line through the origin). A Bland–Altman plot shows the average of two measures on the x-axis and their difference on the y-axis; it allows the evaluation of both biases—that which occurs when the average of the differences between the two paired measurements is significantly different from 0 and the so-called proportional bias that refers to a significant trend between the difference and the magnitude of the measurements, i.e., when the difference in values increases or decreases in proportion to the average values.

## Results

### Inter-laboratory Agreement

As reported in “Materials and Methods,” each institution received from the coordinating center (University of Naples Federico II) a set of 20 cases, providing the coordinator center with the run metric parameters and with the complete list of all the mutations detected and their relative AFs by 10 working days. Then, the samples were aliquoted and shipped to each institution. The mutations detected by the University of Naples Federico II with relative AFs were considered as the gold standard and reported in Table 1. The obtained results, using the SiRe® kit, among all the institutions were fully concordant, reaching an inter-laboratory agreement of 100.00% (Table 1).

### Prospective Evaluation of the SiRe® Panel's Analytical Performance in a Routine Setting

Following the retrospective analysis aimed to assess the inter-laboratory concordance rate, each institution prospectively analyzed a distinct set of 40 cases, including colon or lung cancer tissue samples. All cases (160/160) were successfully analyzed and the success rate was 100.00% (Supplementary Table 2). The median number of reads for the sample was 306,332.38 (ranging from 191 to 976,243), the median number of read length was 128.20 bp (ranging from 60 to 168 bp), the median number of mapped reads was 302,203.01 (ranging from 191 to 908,628), the mean percentage of reads on target was 88.45% (ranging from 14.47 to 99.53%), the average reads for amplicon was 5,380.93 (ranging from seven to 20,385), and the uniformity of coverage was 93.73% (ranging from 71.95 to 100%), in line with the data previously obtained from our group in the “in-house” validation experiments of the SiRe® panel (23).

Regarding the mutant allelic frequencies distribution, a high level of agreement was reached; in particular, the ICC was 0.989 (95% C.I.: 0.981–0.994), and comparing the mutant allelic frequencies distribution with the gold standard, the Linn's concordance correlation coefficient was high for *KRAS* mutation (CCC: 0.977; 95.00% C.I.: 0.963–0.986), *PDGFR*α mutation (CCC: 0.989; 95% C.I.: 0.974–0.996), and *cKIT* mutations (CCC: 0.954; 95% C.I.: 0.888–0.982).

## Discussion

NGS represents a fascinating and versatile technology for the simultaneous analysis of different genes in different cancer patients. However, NGS requires many different laboratory steps, from DNA extraction, libraries preparation, sequencing procedure, and data interpretation, which can lead to results not being always fully consistent and reproducible in different laboratories, a limit inherent to many laboratory-developed tests. Thus, it is widely held that a reliable and cost-effective validation and implementation of this procedure in routine practice would benefit from a high degree of collaboration among skilled and experienced molecular biologists belonging to different institutions. In particular, networking is crucial to meet the challenges related to routine clinical sample processing as, in many cases, issues involve a suboptimal quantity and quality of nucleic acids (15–19). Furthermore, besides driving detection of mutations that are evenly distributed in neoplastic tissue, resistant genomic alteration features heterogeneity in the molecular landscape of many cancers; the detection of distinct mutations in different subclonal neoplastic populations can only be addressed by robust and reliable gene panels, ensuring a uniform coverage of the target regions. In particular, small gene panels, such as the SiRe® NGS panel, filling an intermediate space between allelic-specific PCR approaches and targeted re-sequencing have several advantages (17, 18). In fact, the SiRe® panel has previously been designed and validated for both tissue samples and liquid biopsy specimens to cover 568 clinically relevant mutations in six genes (*EGFR, KRAS, NRAS, BRAF, cKIT*, and *PDGFR*α) involved in NSCLC, gastrointestinal stromal tumor, mCRC, and melanoma, meeting the clinical indication for drug prescription from the European Medical Agency. In the validation study, a high analytical sensitivity (0.005%) with a 0.01% lower limit of detection was reported (19). We also developed a SiRe®-specific preparation protocol to enable the pooling of two 16-sample libraries in each run. Thus, using this well-standardized procedure, we were able to sequence simultaneously up to 32 paired plasma/serum samples in <3 h on the IonTorrent platforms, with a consequent reduction in the total consumable cost, limiting the expense to 98 euro for the simultaneous analysis of six different genes, which is comparable with the cost of the most commercially available real-time PCR-based kits (19–23).

In different European countries, the implementation of NGS in routine diagnostic procedures, beyond pre-analytical and technical factors, strongly depends on more general considerations relative to the healthcare systems in which predictive molecular pathology is practiced. While in North American countries, tumor samples are outsourced in large reference laboratories, thanks to the well-resourced, reimbursement-based systems ensuring the repayment of extensive tumor sequencing. In Italy, NGS is practiced in many different laboratories close to the patients' homes, each of them using different wet and dry procedures. Needless to say, harmonization of different laboratory practices and of mutational databases is strongly needed to improve and homogenize the assessment of genomic biomarkers. To this end, this current study was carried out to assess the feasibility of adopting the same NGS panel in the context of a multi-institution study, thanks to a consortium constituted by five different highly experienced Italian laboratories whose geographical location covers a large part of the Italian peninsula. Our data, generated from the retrospective analysis in each participating laboratory of a set of 20 samples, reached a high interlaboratory agreement level, not only relative to the mutation detection but also in relation to allelic frequencies estimation. Considering the prospective phase of this study, to assess the analytical performance of the SiRe® panel in different routine settings, promising results were obtained; in fact, an overall success rate of 100.00% was reported, with the median values of the run metric parameters confirming that the data obtained in the “in-house” validation data of the SiRe® panel can be successfully reproduced in four different institutions.

The main limitation of the SiRe® NGS panel relies in the limited number of analyzed genes. In particular, as discussed above, in the design and development of our panel, we only focused our attention on the clinically relevant mutations in six genes. In addition, this panel is able to identify point mutations and indel alterations. Thus, further improvements are required to increase the clinical performance of the SiRe® NGS panel. In particular, it is necessary to expand the reference range of the SiRe® NGS gene panel, focusing our attention on other clinically relevant genes and on additional alterations such as copy number variations and gene fusions.

In conclusion, considering altogether the results obtained from the current multi-institution study, the SiRe® NGS panel represents a robust diagnostic tool for mutational analysis in a predictive molecular pathology routine setting, which is useful in harmonizing the NGS procedures in different Italian laboratories.

## Data Availability Statement

The datasets analyzed for this study can be found in the SRA repository (https://www.ncbi.nlm.nih.gov/sra) (PRJNA604200).

## Ethics Statement

Ethical review and approval was not required for the study on human participants in accordance with the local legislation and institutional requirements. The patients/participants provided their written informed consent to participate in this study.

## Author Contributions

UM and GT conceived the study. FP, PP, RSg, MN, CD, RL, RP, GPalo, DV, IB, and RSm performed the experiments and contributed as molecular pathologists. UM, ST, GPalm, MB, PG, LG, and GT supervised the experiments. DB performed the statistical analysis. UM, FP, PP, and GT wrote the manuscript. VC contributed as biostatistic. All of the authors critically reviewed the paper and approved the final version of the manuscript.

### Conflict of Interest

The authors declare that the research was conducted in the absence of any commercial or financial relationships that could be construed as a potential conflict of interest.

## Supplementary Material

Supplementary Table 1 Details relative to the DNA extraction procedures and to the NGS platform employed by participating institutions.

Supplementary Table 2 Run metric parameters of all analyzed samples by participating institutions.

Supplementary File 1 Smart protocol for manual library preparation on Ion Torrent platforms. Permission to publish the protocol was obtained by Genedin s.r.l. (a spin-off of the Department of Public Health, University of Naples Federico II).
