# Supplementary material for: Harmonization of Next-Generation Sequencing Procedure in Italian Laboratories: A Multi-Institutional Evaluation of the SiRe® Panel
Source: Front Oncol. 2020 Mar 11;10:236. doi: 10.3389/fonc.2020.00236 (PMC7078327; doi:10.3389/fonc.2020.00236)
Supplement: Supplementary Table 1 — Details relative to the DNA extraction procedures and to the NGS platform employed by participating institutions. [file Table_1.docx]

**Supplementary Table 1**. Details relative to the DNA extraction procedures and to the NGS platform employed by participating institutions.

| Inst. | Extraction Kit | Extraction protocoll | DNA concentration evaluation | NGS  Platform | Library  Preparation |
| --- | --- | --- | --- | --- | --- |
| DMM | zymoresearch FFPE DNA Miniprep | Manual | Qubit DNA HS | S5Xl | manual |
| IEO | Maxwell RSC DNA FFPE Kit | Automated | Quantus | S5 | manual –  automated |
| ICB | Qiagen Gene ReadDNA FFPE | Manual | Qubit DNA HS | PGM | manual |
| NCI | QIAamp DNA FFPE Tissue Kit | Manual | Qubit DNA HS | PGM | manual |

Abbreviations: Inst. : Istitution; PGM: Personal Genome Machine; DMM: University La Sapienza – Rome; IEO: Istituto Oncologico Europeo – Milan; Istituto ICB: Tumori Giovanni Paolo II – Bari; NCI: Consiglio Nazionale delle Ricerche – Sassari
