# Supplementary material for: Harmonization of Next-Generation Sequencing Procedure in Italian Laboratories: A Multi-Institutional Evaluation of the SiRe® Panel
Source: Front Oncol. 2020 Mar 11;10:236. doi: 10.3389/fonc.2020.00236 (PMC7078327; doi:10.3389/fonc.2020.00236)
Supplement: Supplementary Table 2 — Run metric parameters of all analyzed samples by participating institutions. [file Table_2.docx]

**Supplementary Table 2**. Run metric parameters of all analyzed samples by participating institutions.

|  | Reads | Number of mapped reads | Mean Read Length | Percent reads on target | Average reads per amplicon | Uniformity of amplicon coverage |
| --- | --- | --- | --- | --- | --- | --- |
| DMM 1 | 586374 | 584379 | 124 bp | 83,31% | 116 | 100,00% |
| DMM 2 | 291415 | 290723 | 134 bp | 92,58% | 640 | 97,62% |
| DMM 3 | 505733 | 504070 | 121 bp | 76,38% | 916 | 100,00% |
| DMM 4 | 334423 | 333439 | 131 bp | 86,54% | 680 | 97,62% |
| DMM 5 | 445649 | 444413 | 126 bp | 87,54% | 926 | 100,00% |
| DMM 6 | 444102 | 443186 | 133 bp | 78,93% | 832 | 100,00% |
| DMM 7 | 327986 | 327358 | 135 bp | 87,60% | 682 | 100,00% |
| DMM 8 | 519793 | 518079 | 125 bp | 82,10% | 10,15 | 100,00% |
| DMM 9 | 365327 | 364459 | 129 bp | 80,10% | 6,95 | 100,00% |
| DMM 10 | 493072 | 491351 | 124 bp | 86,09% | 10,11 | 100,00% |
| DMM 11 | 453314 | 452040 | 130 bp | 89,38% | 962 | 97,62% |
| DMM 12 | 274303 | 273979 | 135 bp | 92,19% | 601 | 100,00% |
| DMM 13 | 706243 | 702071 | 121 bp | 88,85% | 1485 | 97,62% |
| DMM 14 | 228912 | 228443 | 136 bp | 91,81% | 499 | 97,62% |
| DMM 15 | 649251 | 645902 | 118 bp | 83,26% | 128 | 100,00% |
| DMM 16 | 586942 | 581461 | 110 bp | 74,44% | 1030 | 100,00% |
| DMM 17 | 161415 | 155500 | 134 bp | 95,82% | 3548 | 92,51% |
| DMM 18 | 155689 | 220750 | 135 bp | 94,60% | 4972 | 92,55% |
| DMM 19 | 221075 | 90543 | 134 bp | 93,62% | 2018 | 92,49% |
| DMM 20 | 90768 | 167886 | 129 bp | 94,62% | 3782 | 92,37% |
| DMM 21 | 168243 | 403992 | 129 bp | 85,52% | 8226 | 92,49% |
| DMM 22 | 405079 | 641757 | 127 bp | 80,44% | 12291 | 92,41% |
| DMM 23 | 644145 | 205959 | 119 bp | 89,52% | 4390 | 92,48% |
| DMM 24 | 206389 | 222439 | 135 bp | 93,77% | 4966 | 92,59% |
| DMM 25 | 222887 | 285733 | 134 bp | 94,25% | 6412 | 91,31% |
| DMM 26 | 286357 | 251508 | 130 bp | 96,47% | 5777 | 92,65% |
| DMM 27 | 251948 | 143496 | 136 bp | 98,16% | 3354 | 92,43% |
| DMM 28 | 143692 | 961765 | 136 bp | 86,88% | 19894 | 92,67% |
| DMM 29 | 976243 | 135755 | 122 bp | 85,88% | 2776 | 90,91% |
| DMM 30 | 671700 | 668339 | 128 bp | 96,81% | 15405 | 90,68% |
| DMM 31 | 448562 | 447493 | 132 bp | 95,52% | 10177 | 90,88% |
| DMM 32 | 889066 | 868545 | 122 bp | 94,04% | 19447 | 92,23% |
| DMM 33 | 614273 | 610629 | 124 bp | 90,13% | 13103 | 92,21% |
| DMM 34 | 507279 | 506786 | 140 bp | 95,49% | 11523 | 90,84% |
| DMM 35 | 592875 | 585537 | 122 bp | 88,61% | 12354 | 90,58% |
| DMM 36 | 441916 | 437363 | 124 bp | 92,81% | 9665 | 90,86% |
| DMM 37 | 374047 | 371892 | 125 bp | 81,18% | 7188 | 91,04% |
| DMM 38 | 412473 | 411708 | 137 bp | 91,41% | 8961 | 90,56% |
| DMM 39 | 210758 | 210469 | 133 bp | 98,75% | 4948 | 90,40% |
| DMM 40 | 599254 | 594065 | 124 bp | 89,32% | 12634 | 92,11% |
| DMM 41 | 473206 | 408734 | 110 bp | 67,38% | 6014 | 92,53% |
| DMM 42 | 138894 | 106654 | 131 bp | 89,37% | 1529 | 92,69% |
| DMM 43 | 206389 | 160870 | 129 bp | 90,30% | 2539 | 90,92% |
| DMM 44 | 281618 | 239889 | 133 bp | 91,87% | 1025 | 92,33% |
| DMM 45 | 423483 | 338441 | 131 bp | 93,27% | 1801 | 92,49% |
| DMM 46 | 376649 | 268382 | 120 bp | 81,11% | 6581 | 92,75% |
| DMM 47 | 71915 | 57077 | 128 bp | 90,44% | 25679 | 91,12% |
| DMM 48 | 118218 | 93927 | 97 bp | 61,55% | 5604 | 93,05% |
| DMM 49 | 46881 | 37909 | 105 bp | 64,72% | 9048 | 92,77% |
| DMM 50 | 81153 | 67379 | 126 bp | 85,33% | 2204 | 92,59% |
| DMM 51 | 341847 | 277853 | 134 bp | 92,18% | 3342 | 91,02% |
| DMM 52 | 1193991 | 958458 | 129 bp | 90,70% | 2380 | 92,39% |
| DMM 53 | 391826 | 260220 | 125 bp | 87,40% | 8348 | 91,20% |
| DMM 54 | 592147 | 400961 | 130 bp | 93,24% | 6302 | 92,61% |
| DMM 55 | 108682 | 83629 | 130 bp | 92,63% | 3948 | 91,20% |
| DMM 56 | 152460 | 124768 | 137 bp | 92,24% | 843,8 | 92,56% |
| DMM 57 | 110354 | 88518 | 142 bp | 88,22% | 2669 | 92,03% |
| DMM 58 | 402966 | 320335 | 146 bp | 88,68% | 2929 | 92,51% |
| DMM 59 | 284401 | 234445 | 145 bp | 90,39% | 2958 | 92,41% |
| DMM 60 | 179202 | 146586 | 139 bp | 92,59% | 3554 | 92,57% |
| IEO 1 | 65015 | 64855 | 149 bp | 84,11% | 1299 | 92,23% |
| IEO 2 | 127746 | 127503 | 129 bp | 93,11% | 2827 | 92,13% |
| IEO 3 | 187207 | 186886 | 131 bp | 93,92% | 4179 | 91,54% |
| IEO 4 | 172323 | 171873 | 120 bp | 85,16% | 3485 | 91,95% |
| IEO 5 | 260038 | 259007 | 115 bp | 84,66% | 5221 | 92,29% |
| IEO 6 | 157316 | 157081 | 130 bp | 94,72% | 3543 | 92,05% |
| IEO 7 | 302191 | 301299 | 124 bp | 88,54% | 6352 | 92,41% |
| IEO 8 | 218418 | 217636 | 127 bp | 89,05% | 4615 | 92,15% |
| IEO 9 | 133241 | 132991 | 137 bp | 92,25% | 2921 | 92,71% |
| IEO 10 | 128940 | 128706 | 137 bp | 93,05% | 2852 | 92,21% |
| IEO 11 | 162923 | 162633 | 135 bp | 94,56% | 3662 | 92,13% |
| IEO 12 | 190321 | 189778 | 120 bp | 91,57% | 4138 | 92,07% |
| IEO 13 | 98747 | 92625 | 77 bp | 45,15% | 995,7 | 92,25% |
| IEO 14 | 279712 | 275553 | 90 bp | 62,72% | 4115 | 87,43% |
| IEO 15 | 274103 | 273154 | 125 bp | 93,90% | 6107 | 92,15% |
| IEO 16 | 266193 | 246798 | 114 bp | 92,39% | 5429 | 89,34% |
| IEO 17 | 13284 | 132549 | 136 bp | 95,95% | 3028 | 91,97% |
| IEO 18 | 227157 | 226091 | 122 bp | 95,90% | 5162 | 88,37% |
| IEO 19 | 142697 | 123941 | 81 bp | 35,05% | 1034 | 91,67% |
| IEO 20 | 20054 | 200229 | 146 bp | 95,22% | 4539 | 92,61% |
| IEO 21 | 195885 | 195224 | 129 bp | 90,80% | 4220 | 90,96% |
| IEO 22 | 306260 | 303716 | 103 bp | 80,88% | 5848 | 84,46% |
| IEO 23 | 25905 | 22108 | 93 bp | 27,35% | 144 | 88,13% |
| IEO 24 | 163740 | 163527 | 168 bp | 95,84% | 3731 | 92,29% |
| IEO 25 | 182439 | 181698 | 126 bp | 95,72% | 4141 | 89,98% |
| IEO 26 | 52377 | 43571 | 71 bp | 67,20% | 697,1 | 80,43% |
| IEO 27 | 235275 | 234648 | 135 bp | 93,75% | 5238 | 91,87% |
| IEO 28 | 164946 | 164352 | 133 bp | 92,02% | 3601 | 92,15% |
| IEO 29 | 362365 | 361869 | 143 bp | 92,59% | 7263 | 93,57% |
| IEO 30 | 353225 | 352997 | 131 bp | 94,78% | 7170 | 90,84% |
| IEO 31 | 369767 | 369458 | 128 bp | 94,24% | 7544 | 93,86% |
| IEO 32 | 319987 | 319680 | 130 bp | 94,22% | 6520 | 93,45% |
| IEO 33 | 342972 | 342435 | 131 bp | 94,10% | 6995 | 93,25% |
| IEO 34 | 195411 | 194946 | 128 bp | 94,91% | 3978 | 93,43% |
| IEO 35 | 203168 | 202737 | 135 bp | 95,28% | 4199 | 93,31% |
| IEO 36 | 359278 | 358921 | 127 bp | 95,49% | 7374 | 93,69% |
| IEO 37 | 451452 | 450943 | 129 bp | 94,67% | 9147 | 93,78% |
| IEO 38 | 260958 | 260496 | 132 bp | 95,24% | 5375 | 94,00% |
| IEO 39 | 426452 | 425869 | 130 bp | 95,00% | 8724 | 94,00% |
| IEO 40 | 514728 | 514220 | 128 bp | 94,90% | 10526 | 94,00% |
| IEO 41 | 321777 | 238804 | 132 bp | 85,85% | 6543 | 91,55% |
| IEO 42 | 132442 | 105512 | 119 bp | 81,15% | 18702 | 92,29% |
| IEO 43 | 249275 | 202371 | 135 bp | 91,71% | 5434 | 90,78% |
| IEO 44 | 288330 | 237129 | 128 bp | 94,03% | 6444 | 91,76% |
| IEO 45 | 518025 | 358430 | 106 bp | 76,02% | 9186 | 91,89% |
| IEO 46 | 550636 | 413591 | 115 bp | 82,62% | 10791 | 91,97% |
| IEO 47 | 139605 | 114916 | 124 bp | 88,46% | 8953 | 92,13% |
| IEO 48 | 122263 | 104790 | 111 bp | 72,39% | 24558 | 92,79% |
| IEO 49 | 362365 | 300371 | 116 bp | 76,99% | 9849 | 91,97% |
| IEO 50 | 353225 | 294463 | 130 bp | 89,96% | 8381 | 92,15% |
| IEO 51 | 188834 | 153569 | 132 bp | 96,32% | 16409 | 90,78% |
| IEO 52 | 426561 | 334103 | 101 bp | 76,30% | 15222 | 88,29% |
| IEO 53 | 1441992 | 923042 | 74 bp | 41,18% | 9437 | 87,05% |
| IEO 54 | 539979 | 384713 | 102 bp | 81,37% | 15069 | 90,52% |
| IEO 55 | 392384 | 310141 | 113 bp | 88,26% | 24008 | 88,92% |
| IEO 56 | 716804 | 603278 | 138 bp | 87,66% | 20312 | 92,15% |
| IEO 57 | 844905 | 627749 | 140 bp | 93,27% | 4132 | 90,62% |
| IEO 58 | 991868 | 699728 | 147 bp | 91,14% | 2869 | 90,94% |
| IEO 59 | 783757 | 594486 | 142 bp | 92,70% | 4161 | 90,86% |
| IEO 60 | 1147565 | 909360 | 137 bp | 89,88% | 7932 | 92,01% |
| ICB 1 | 407438 | 404738 | 129 bp | 88,14% | 8,494 | 97,62% |
| ICB 2 | 200330 | 199908 | 145 bp | 92,52% | 4404 | 97,62% |
| ICB 3 | 714297 | 708986 | 118 bp | 77,33% | 13053 | 97,62% |
| ICB 4 | 271295 | 270489 | 137 bp | 94,76% | 6103 | 97,62% |
| ICB 5 | 191,893 | 191,475 | 131 bp | 94,88% | 4326 | 100,00% |
| ICB 6 | 515947 | 511480 | 121 bp | 95,43% | 11621 | 100,00% |
| ICB 7 | 162879 | 162528 | 131 bp | 96,69% | 3742 | 100,00% |
| ICB 8 | 140197 | 139584 | 126 bp | 91,33% | 3035 | 97,62% |
| ICB 9 | 428402 | 406747 | 60 bp | 14,47% | 1401 | 88,10% |
| ICB 10 | 182945 | 182428 | 136 bp | 95,09% | 413 | 97,62% |
| ICB 11 | 234537 | 233515 | 134 bp | 79,76% | 5084 | 100,00% |
| ICB 12 | 19179 | 19116 | 135 bp | 95,44% | 434,4 | 100,00% |
| ICB 13 | 98075 | 97887 | 130 bp | 94,22% | 2196 | 100,00% |
| ICB 14 | 390936 | 387866 | 124 bp | 80,64% | 7447 | 100,00% |
| ICB 15 | 353670 | 351741 | 130 bp | 93,96% | 7869 | 97,62% |
| ICB 16 | 81971 | 81779 | 142 bp | 93,40% | 1819 | 97,62% |
| ICB 17 | 88142 | 87933 | 145 bp | 95,35% | 1996 | 97,62% |
| ICB 18 | 243438 | 242,157 | 126 bp | 81,28% | 4686 | 100,00% |
| ICB 19 | 894844 | 890886 | 129 bp | 96,10% | 20385 | 97,62% |
| ICB 20 | 136354 | 135356 | 138 bp | 56,13% | 1809 | 100,00% |
| ICB 21 | 276201 | 253762 | 143 bp | 95,01% | 574 | 97,62% |
| ICB 22 | 333077 | 332151 | 129 bp | 92,78% | 7337 | 97,62% |
| ICB 23 | 19871 | 19849 | 143 bp | 97,31% | 459,9 | 95,24% |
| ICB 24 | 115853 | 115555 | 155 bp | 91,99% | 2531 | 100,00% |
| ICB 25 | 47502 | 44303 | 155 bp | 93,96% | 991,2 | 100,00% |
| ICB 26 | 580741 | 578954 | 130 bp | 94,83% | 13,072 | 97,62% |
| ICB 27 | 76201 | 76069 | 143 bp | 94,18% | 1706 | 97,62% |
| ICB 28 | 142477 | 142119 | 132 bp | 94,92% | 3212 | 97,62% |
| ICB 29 | 12708 | 12684 | 143 bp | 95,09% | 287,2 | 97,62% |
| ICB 30 | 128756 | 128364 | 125 bp | 89,33% | 273 | 100,00% |
| ICB 31 | 230474 | 223792 | 94 bp | 63,96% | 3096 | 97,62% |
| ICB 32 | 443423 | 438967 | 103 bp | 61,70% | 6449 | 100,00% |
| ICB 33 | 68206 | 68047 | 137 bp | 88,99% | 1442 | 97,62% |
| ICB 34 | 401699 | 400492 | 125 bp | 94,86% | 9045 | 100,00% |
| ICB 35 | 333077 | 332151 | 129 bp | 92,78% | 7337 | 97.62% |
| ICB 36 | 549944 | 545850 | 126 bp | 80,28% | 10434 | 100,00% |
| ICB 37 | 233715 | 231733 | 136 bp | 86,00% | 4745 | 97,62% |
| ICB 38 | 932530 | 908628 | 112 bp | 78,91% | 17072 | 100,00% |
| ICB 39 | 400419 | 396064 | 116 bp | 81,38% | 7674 | 100,00% |
| ICB 40 | 10362 | 103306 | 138 bp | 88,68% | 2181 | 95,24% |
| ICB 41 | 531788 | 402892 | 121 bp | 83,01% | 10399 | 100,00% |
| ICB 42 | 68026 | 55499 | 100 bp | 68,02% | 102 | 97,62% |
| ICB 43 | 887521 | 659755 | 93 bp | 75,68% | 13656 | 100.00% |
| ICB 44 | 164400 | 130428 | 111 bp | 76,39% | 20555 | 97.62% |
| ICB 45 | 762939 | 544275 | 37 bp | 46,17% | 4971 | 19,05% |
| ICB 46 | 1007495 | 758274 | 117 bp | 80,93% | 19246 | 100,00% |
| ICB 47 | 140660 | 108222 | 148 bp | 88,96% | 2972 | 97,62% |
| ICB 48 | 535948 | 410805 | 125 bp | 83,55% | 10599 | 97,62% |
| ICB 49 | 170812 | 135212 | 158 bp | 92,19% | 3743 | 97,62% |
| ICB 50 | 220674 | 171235 | 161 bp | 91,90% | 4819 | 97,62% |
| ICB 51 | 81971 | 66796 | 156 bp | 95,11% | 2733 | 97,62% |
| ICB 52 | 401699 | 346876 | 143 bp | 84,80% | 11702 | 97,62% |
| ICB 53 | 378479 | 303311 | 145 bp | 85,15% | 6472 | 97,62% |
| ICB 54 | 140197 | 113160 | 148 bp | 90,19% | 7094 | 100,00% |
| ICB 55 | 19871 | 17055 | 135 bp | 90,67% | 10629 | 100,00% |
| ICB 56 | 253535 | 200390 | 153 bp | 89,19% | 5531 | 97,62% |
| ICB 57 | 44394 | 38192 | 164 bp | 91,57% | 275 | 97,62% |
| ICB 58 | 78988 | 67623 | 162 bp | 86,04% | 3467 | 97,62% |
| ICB 59 | 162879 | 141120 | 156 bp | 91,42% | 8225 | 97,62% |
| ICB 60 | 493779 | 396917 | 135 bp | 91,02% | 6446 | 95,24% |
| NCI 1 | 190275 | 189763 | 132 bp | 94,75% | 4281 | 91,20% |
| NCI 2 | 136995 | 136760 | 136 bp | 95,92% | 3123 | 90,70% |
| NCI 3 | 169286 | 168899 | 134 bp | 92,55% | 3722 | 91,04% |
| NCI 4 | 253463 | 252975 | 130 bp | 93,18% | 5612 | 90,98% |
| NCI 5 | 243128 | 242547 | 131 bp | 88,35% | 5102 | 86,27% |
| NCI 6 | 492548 | 428528 | 136 bp | 92,17% | 9404 | 92,13% |
| NCI 7 | 371064 | 364150 | 129 bp | 94,02% | 8152 | 89,12% |
| NCI 8 | 434929 | 432779 | 127 bp | 94,30% | 9717 | 91,81% |
| NCI 9 | 495525 | 494922 | 139 bp | 97,40% | 11477 | 71,95% |
| NCI 10 | 494789 | 494105 | 137 bp | 94,29% | 11093 | 90,94% |
| NCI 11 | 620175 | 619367 | 146 bp | 92,00% | 13567 | 90,84% |
| NCI 12 | 428405 | 427939 | 136 bp | 94,60% | 9639 | 88,47% |
| NCI 13 | 374556 | 372867 | 115 bp | 74,12% | 6580 | 91,96% |
| NCI 14 | 314782 | 314267 | 137 bp | 95,75% | 7165 | 90,62% |
| NCI 15 | 432558 | 431729 | 133 bp | 96,15% | 9884 | 90,62% |
| NCI 16 | 314969 | 314071 | 134 bp | 93,28% | 6976 | 91,97% |
| NCI 17 | 251195 | 250786 | 151 bp | 93,25% | 5568 | 91,97% |
| NCI 18 | 114962 | 114585 | 152 bp | 91,45% | 2495 | 91,14% |
| NCI 19 | 133784 | 133365 | 149 bp | 88,87% | 2822 | 91,08% |
| NCI 20 | 175673 | 175212 | 137 bp | 93,54% | 3902 | 91,09% |
| NCI 21 | 325861 | 325082 | 137 bp | 93,04% | 7201 | 92,31% |
| NCI 22 | 562830 | 561611 | 132 bp | 94,19% | 12595 | 92,13% |
| NCI 23 | 664277 | 662648 | 127 bp | 93,57% | 14763 | 91,97% |
| NCI 24 | 610556 | 608965 | 127 bp | 93,41% | 13544 | 92,49% |
| NCI 25 | 372172 | 371521 | 132 bp | 98,27% | 8693 | 90,76% |
| NCI 26 | 81278 | 81134 | 140 bp | 95,67% | 1848 | 90,76% |
| NCI 27 | 117264 | 117029 | 139 bp | 92,66% | 2582 | 90,76% |
| NCI 28 | 210260 | 209861 | 130 bp | 91,51% | 4573 | 92,13% |
| NCI 29 | 159625 | 159393 | 136 bp | 91,17% | 3460 | 90,76% |
| NCI 30 | 201283 | 200890 | 132 bp | 93,49% | 4472 | 92,15% |
| NCI 31 | 219673 | 219247 | 128 bp | 90,99% | 4750 | 92,13% |
| NCI 32 | 167682 | 167331 | 135 bp | 94,28% | 3756 | 90,76% |
| NCI 33 | 136222 | 136011 | 137 bp | 92,09% | 2982 | 86,56% |
| NCI 34 | 199683 | 199165 | 133 bp | 89,76% | 4256 | 90,56% |
| NCI 35 | 241854 | 240866 | 124 bp | 88,77% | 5091 | 90,58% |
| NCI 36 | 212074 | 211417 | 129 bp | 88,87% | 4474 | 90,73% |
| NCI 37 | 48724 | 486569 | 117 bp | 99,53% | 5264 | 94,32% |
| NCI 38 | 501589 | 500409 | 130 bp | 91,94% | 10954 | 90,96% |
| NCI 39 | 336123 | 333709 | 110 bp | 50,00% | 3604 | 87,80% |
| NCI 40 | 475598 | 473079 | 118 bp | 70,00% | 7841 | 86,27% |
| NCI 41 | 209434 | 155982 | 119 bp | 80,54% | 3992 | 89,86% |
| NCI 42 | 184842 | 116028 | 98 bp | 49,32% | 2152 | 77,95% |
| NCI 43 | 88677 | 63241 | 154 bp | 78,86% | 1658 | 90,76% |
| NCI 44 | 213798 | 151354 | 158 bp | 78,23% | 3964 | 92,29% |
| NCI 45 | 207768 | 130005 | 114 bp | 66,50% | 3255 | 92,25% |
| NCI 46 | 177345 | 132203 | 134 bp | 80,50% | 3382 | 91,12% |
| NCI 47 | 102793 | 78543 | 150 bp | 86,13% | 2100 | 90,98% |
| NCI 48 | 68153 | 53268 | 158 bp | 88,42% | 1431 | 91,06% |
| NCI 49 | 63393 | 49757 | 177 bp | 90,25% | 1358 | 91,59% |
| NCI 50 | 79229 | 61092 | 154 bp | 88,00% | 1654 | 91,32% |
| NCI 51 | 210568 | 148969 | 121 bp | 75,63% | 3774 | 92,39% |
| NCI 52 | 147877 | 112800 | 131 bp | 84,80% | 2971 | 91,15% |
| NCI 53 | 241506 | 193788 | 119 bp | 87,86% | 5032 | 92,43% |
| NCI 54 | 252345 | 203244 | 121 bp | 88,32% | 5279 | 92,35% |
| NCI 55 | 128938 | 108139 | 138 bp | 94,05% | 2878 | 92,27% |
| NCI 56 | 494789 | 432844 | 128 bp | 95,62% | 5098 | 90,96% |
| NCI 57 | 325861 | 274531 | 130 bp | 94,20% | 5098 | 90,62% |
| NCI 58 | 241854 | 201263 | 134 bp | 93,08% | 3461 | 90,44% |
| NCI 59 | 114962 | 95050 | 128 bp | 93,29% | 4964 | 90,42% |
| NCI 60 | 243128 | 197692 | 129 bp | 93,48% | 5372 | 90,60% |

Abbreviations: DMM: University La Sapienza – Rome; IEO: Istituto Oncologico Europeo – Milan; Istituto ICB: Tumori Giovanni Paolo II – Bari; NCI: Consiglio Nazionale delle Ricerche – Sassari
