# Supplementary material for: Harmonization of Next-Generation Sequencing Procedure in Italian Laboratories: A Multi-Institutional Evaluation of the SiRe® Panel
Source: Front Oncol. 2020 Mar 11;10:236. doi: 10.3389/fonc.2020.00236 (PMC7078327; doi:10.3389/fonc.2020.00236)
Supplement: Supplementary File 1 — Smart protocol for manual library preparation on Ion Torrent platforms. Permission to publish the protocol was obtained by Genedin s.r.l. (a spin-off of the Department of Public Health, University of Naples Federico II). [file Data_Sheet_1.pdf]

Smart Protocol for manual library preparation on Ion Torrent platforms

SiRe ®

For Tissue samples

Before starting:

- Quantify genomic DNA and eventually dilute at 20 ng/µl. If DNA concentration is < 20 ng/µl add a DNA input from 2 to 6 µL.
- Before the use equilibrate reagents at room temperature for 20 min.
- Briefly vortex and spin the reagents.

Amplification Step

- Prepare a PCR reaction following the table:

**Reagents**	**Volume**	**Storage temperature**
Master Mix	4 µL	–30ºC to –10ºC
SiRe® (2X)	10 µL	–30ºC to –10ºC
DNA	1-6 µL	–30ºC to –10ºC
Nuclease-free Water	6 – DNA volume (µL)	T.A.

- To amplify target regions, run the following program

**Stage**	**Step**	**Temperature**	**Time**
Hold	Activate enzyme	99°C	2 min
Cicles (26)	Denaturate	99°C	15 sec
	Anneal and extend	60°C	4 min
Hold		10°C	Hold

Endonuclease digestion

NOTE: Enzyme Reagent is viscous. Pipet slowly and mix thoroughly. Perform this step on ice or a cold block, then quickly proceed to incubation.

- Spin the samples;
- Add 2 µl di endonuclease to each amplfied sample. The total volume is ~22 µL.
- load in the thermal cycler, then run the following program:

Temperature	Time
50°C	10 min
55°C	10 min
60°C	20 min
10°C	≤ 1 h

- Store the samples at 10°C before ≤ 1 h of the last step

Ligate adapters to the amplicons and purify (not included in ***SiRe*** *® Kit)*

- For each barcode X selected, prepare a mix of Ion P1 Adapter and Ion Xpress™ Barcode X (not provided) at a final dilution of 1:4 for each adapter. For example, combine the volumes indicated in the following table. Scale volumes as necessary. Use 2 µL of this barcode adapter mix in step below. (For any detail refer to Thermofisher specific manual)

**Reagents**	**Volume**
Ion P1 Adapter	2 µL
Ion Xpress™ Barcode X	2 µL
Nuclease-free Water	4 µL

NOTE: Store diluted adapters at –20°C. Barcode diluted must be used in 30 days.

Vortex and centrifuge Switch solution and diluted barcodes; only centrifuge DNA ligase.

Add reagents as follows:

**Order of addition**	**Reagents**	**Volume**
1°	Switch Solution	4 µL
2°	Barcodes X	2 µL
3°	DNA Ligasi	2 µL

NOTE: Total volume (including ~22 µL of digested amplicon).

- Vortex and spin the samples;
- Load in the thermal cycler, then run the following program:

**Temperature**	**Time**
22°C	30 min
68°C	5 min
72°C	5 min
10°C	≤ 1 h

- Samples can be stored for up to 24 hours at 10°C on the thermal cycler. For longer periods, store at –20°C.

Libraries purification

Prepare two falcons of 10 ml freshly prepared 70% ethanol

- add 45 µL (1.5X sample volume) of Agencourt™ AMPure™ XP Reagent (not provided) to each library. Pipet up and down 5 times to mix the bead suspension with the DNA thoroughly.
- Incubate the mixture for 5 minutes at room temperature.
- Place the tubes in a magnetic rack, then incubate for 2 minutes or until solution clears. Carefully remove, then discard the supernatant without disturbing the pellet.
- Add 150 µL of freshly prepared 70% ethanol, then move the plate side-to-side in the two positions of the magnet to wash the beads. Carefully remove, then discard the supernatant without disturbing the pellet.
- Repeat step 5 for a second wash
- Ensure that all ethanol droplets are removed from the wells. Keeping the tubes in the magnet, air-dry the beads at room temperature for 5 minutes. Do not overdry.

NOTE: Dilute Agencourt™ AMPure™XP in a low bind 1,5 ml tube and store at+4°C, before the use equilibrate at R.T , vortex 1 min and centrifuge.

NOTE: Agencourt™ AMPure™XP, ethanol are not included in the kit.

Library Amplification

- Remove the samples with purified libraries from the magnetic rack, then add 50 µL of Low TE to the pellet to disperse the beads.
- Transfer samples in magnetic rack for 5 min.

- Transfer 50 µl of supernatant in a new 0,2 ml tube. centrifuge, load in the thermal cycler, then run the following program :

**Stage**	**Temperature**	**Time**
Hold	98°C	2 min
5 Cicli	98°C	15 sec
	60°C	1 min
Hold	10°C	∞

NOTE: Stopping point. It is possible to store samples at 20°C for 30 days

- Perform a two-round purification process with the Agencourt™ AMPure™ XP Reagent:
- Transfer samples in a new low bind 1,5 ml tube.
- Add 25 µL (0.5X sample volume) of Agencourt™ AMPure™ XP Reagent to each sample containing ~50 µL of sample. Mix the bead suspension with the DNA thoroughly by pipetting up and down 5 times.
- Incubate the mixture for 5 minutes at room temperature.
- Place the samples in a magnet for at least 5 minutes, or until the solution is clear.
- Carefully transfer the supernatant from each tube to a new low bind 1,5 ml tube without disturbing the pellet.
- To the supernatant add 60 µL (1.2X original sample volume) of Agencourt™ AMPure™ XP Reagent. Pipet up and down 5 times to mix the bead suspension with the DNA thoroughly.
- Incubate the mixture for 5 minutes at room temperature
- Place the samples in the magnetic rack for 3 minutes or until the solution is clear. Carefully remove, then discard the supernatant without disturbing the pellet.
- Add 150 µL of freshly prepared 70% ethanol to each tube, then move the tubes side to side in the magnet to wash the beads. Remove, then discard the supernatant without disturbing the pellet.
- Repeat this step for a second wash removing supernatant.
- Ensure that all ethanol droplets are removed from the tubes. Keeping the tubes in the magnet, air-dry the beads at room temperature for 5 minutes. Do not overdry.
- Remove the tubes from the magnet, then add 50 µL of Low TE to the pellet to disperse the beads.
- Keeping tubes in magnet, transfer supernatant in a new low binding tube 1,5 ml.

Suggested - Qubit™ Fluorometer: Quantify the library and calculate the dilution factor

- Analyze 10 µL of each ampl□fied library using a Qubit™ Fluorometer and the Qubit™ dsDNA HS Assay Kit.
- Combine 10 µL of the ampl□fied Ion AmpliSeq™ library with 190 µL of dye reagent, mix well, then incubate for at least 2 minutes in a 0,5 ml tube.
- Measure the concentration on the Qubit™ Fluorometer.
- Calculate the concentration of the undiluted library by multiplying by 20
- Based on the calculated library concentration, determine the dilution that results in a concentration of ~100 pM:

NOTE: STOPPING POINT. Libraries can be stored at +4 °C for 15 days

(Please to proceed refer to specific platform manual)
